# Feasibility and accuracy of ED frailty identification in older trauma patients: a prospective multi-centre study

**DOI:** 10.1186/s13049-021-00868-4

**Published:** 2021-03-30

**Authors:** Heather Jarman, Robert Crouch, Mark Baxter, Chao Wang, George Peck, Dhanupriya Sivapathasuntharam, Cara Jennings, Elaine Cole

**Affiliations:** 1 Emergency Department Clinical Research Unit, St George’s University Hospitals NHS Foundation Trust, Blackshaw Road, London, SW17 0QT UK; 2 University Hospital Southampton NHS Foundation Trust, Southampton, UK; 3 Faculty of Health, Social Care and Education, Kingston University and St George’s, University of London, London, UK; 4 Imperial College Healthcare NHS Trust, London, UK; 5 Bart’s Health NHS Trust, London, UK; 6 King’s College Hospital NHS Foundation Trust, London, UK; 7 Blizard Institute, Queen Mary’s, University of London, London, UK

**Keywords:** Frailty, Major trauma, Older people, Nursing

## Abstract

**Background:**

The burden of frailty on older people is identifiable by its adverse effect on mortality, morbidity and long term functional and health outcomes. In patients suffering from a traumatic injury there is increasing evidence that it is frailty rather than age that impacts greatest on these outcomes and that early identification can guide frailty specific care. The aim of this study was to evaluate the feasibility of nurse-led assessment of frailty in older trauma patients in the ED in patients admitted to major trauma centres.

**Methods:**

Patients age 65 years and over attending the Emergency Departments (ED) of five Major Trauma Centres following traumatic injury were enrolled between June 2019 and March 2020. Patients were assessed for frailty whilst in the ED using three different screening tools (Clinical Frailty Scale [CFS], Program of Research to Integrate Services for the Maintenance of Autonomy 7 [PRIMSA7], and the Trauma Specific Frailty Index [TSFI]) to compare feasibility and accuracy. Accuracy was determined by agreement with geriatrician assessment of frailty. The primary outcome was identification of frailty in the ED using three different assessment tools.

**Results:**

We included 372 patients whose median age was 80, 53.8% of whom were female. The most common mechanism of injury was fall from less than 2 m followed by falls greater than 2 m. Completion rates for the tools were variable, 31.9% for TSFI, compared to 93% with PRISMA7 and 98.9% with the CFS. There was substantial agreement when using CFS between nurse defined frailty and geriatrician defined frailty. Agreement was moderate using PRISMA7 and slight using TSFI.

**Conclusions:**

This prospective study has demonstrated that screening for frailty in older major trauma patients within the Emergency Department is feasible and accurate using CFS.

**Trial registration:**

ISRCTN, ISRCTN10671514. Registered 22 October 2019

**Supplementary Information:**

The online version contains supplementary material available at 10.1186/s13049-021-00868-4.

## Introduction

### Background

Frailty is a condition characterised by a cumulative decline of physiological resilience across several body systems [1–3]. The ageing population is increasing and so is the prevalence of frailty, with estimates ranging from 4.0–59.1% [4].

In the UK, national trauma registry data show that more older people are sustaining major trauma, with a rise in those aged 75 and over from 8.1% of cases in 1990 to 53.8% in 2013 [5]. Older trauma patients are more likely to suffer adverse outcomes compared to younger patients despite similar injury severity [6, 7]. Frailty has been linked to worse outcomes, such as longer hospital stay and mortality in a variety of clinical situations, including in emergency surgery and patients with fractured neck of femur [8, 9]. Considering older people as one population may be misleading due to heterogeneity in pre-injury functional status, comorbidities and physiologic condition. Although there is overlap with multi-morbidity and chronological age, frailty is different. Older trauma patients fall into one of two groups – those who are functioning well prior to injury, and those with more complex health needs, sometimes referred to as geriatric syndromes, including frailty. It is this pre-injury frailty, as well as age, that appears to influence outcome with those who are frail suffering worse outcomes and increased mortality [10, 11].

There are broadly two overlapping models of frailty, the cumulative deficit model and the phenotype model [12, 13]. The cumulative deficit model considers frailty as a number of ‘deficits’ (variables including symptoms, disease states and abnormal laboratory findings) where the more variables that a person has the more likely that they are frail [12]. The frailty phenotype model presents five variables associated with frailty: unintentional weight loss, self-reported exhaustion, low energy expenditure, slow gait speed, weak grip strength [13]. Key to both these models is frail patients are at risk of significant functional, physical and cognitive decline following an episode of illness or injury [4. Clegg]. Despite the characterisation of frailty in these models, patients with frailty represent a heterogeneous group requiring individual adaptations to their assessment and treatment. This makes recognition of frailty in the emergency care environment challenging.

There is increasing recognition of the benefit of early identification of frailty to predict outcome or guide resource use in older emergency surgery and trauma patients [14, 15]. Despite this there is a lack of consensus of how and when frailty should be identified in patients with major traumatic injuries [16]. In the UK, the British Geriatric Society makes recommendation that frailty assessment occurs across all healthcare settings and in patients with different clinical conditions but do not recommend a specific tool for use in major trauma [1].

The identification of major trauma patients who are frail or are at risk of frailty should lead to frailty specific major trauma pathways initiated in the ED which may lead to an improvement in patient outcomes. However, a recent international scoping review reported only 14% of patients were frailty screened during this phase of care [17], and a systematic mapping review found a lack of consensus evidence on how to identify frail older people in the ED [18]. In UK Major Trauma Centres a quality measure leading to a payment subsidy has recently been introduced requiring that all patients aged ≥65 years have a Clinical Frailty Scale completed within 72 h of admission by a geriatrician rather than within the ED [19]. The timing of this assessment at this stage in the admission process has been designed to promote diversion of geriatrician resource to major trauma patients, but may not be optimum in providing early frailty specific care to those most in need and earlier identification could lead to better targeting of multi-disciplinary resource.

The prevalence of frailty in the UK major trauma population is not currently known, nor do we know whether it is feasible to carry out accurate frailty assessment in the ED in this patient group. To address this, we performed a prospective study to determine the accuracy of frailty assessment undertaken by ED nurses using three scoring tools against the reference standard of a geriatrician assessment (GA). The overall aim was to evaluate the feasibility of nurse-led assessment of frailty in the ED in patients aged 65 years or over admitted to major trauma centres. Primarily we aimed to assess and compare the performance of different tools in identifying frailty. We also sought to determine the prevalence of frailty in this population of trauma patients, and examine the outcomes associated with frailty.

## Methods

### Study design

This is a prospective observational study carried out between June 2019 and March 2020. The methods of this study have been previously published [20]. The study was approved by the UK Social Care Research Ethics Committee (REC no 19/IEC08/0006) in March 2019, trial registration number: ISRCTN12345678. The study was prospectively registered on the National Institute for Health Research (NIHR) portfolio (reference UK CRN 41047).

### Setting

The study was carried out at five Major Trauma Centres (Level 1 equivalent hospitals) in the south of England. The population covered by the study was approximately 2.2 million people, with characteristics of each of the MTCs shown in Table 1. The EDs treated a total of 714,655 patients in 2019.

**Table 1:** Characteristics of sites

Major Trauma Centre	Trauma population served	Total ED attendances 2019^a^	Total trauma team activations based on hospital criteria 2019	Total team activations based on hospital criteria ≥65y 2019
Kings College Hospital	5 million	186, 137	2142	393
Royal London Hospital	4.3 million	128, 904	3095	490
Southampton University Hospital	3.5 million	116, 010	594^b^	Not available
St George’s Hospital	3.5 million	161, 369	2407	692
St Mary’s Hospital	3.9 million	122, 235	3032	692

Data source: local hospital data except ^a^ from https://www.england.nhs.uk/statistics/statistical-work-areas/ae-waiting-times-and-activity. ^b^ TARN eligible patients only

### Participants

Patients were eligible to participate if they were aged 65 or over, required activation of the trauma team at the receiving hospital and were subsequently admitted. Patients were enrolled if they met the eligibility criteria and there was a nurse trained to consent and use the frailty assessment tools available. Patients who were unable to consent to take part in the study initially due to injury or existing cognitive impairment were enrolled using consultee consent procedures and subsequently withdrawn if patient or next of kin consent could not be gained.

All study data were prospectively collected from either patient or relative information or the clinical records by a research nurse using a standardised reporting form. Patients were anonymised and identified using a study identification number. Data were uploaded to a secure online database, REDCap (Research Electronic Data Capture, Vanderbilt University hosted by St George’s, University of London).

### Variables

The primary outcome is identification of frailty. Secondary outcomes included in-hospital mortality, critical care and hospital length of stay and discharge to the usual place of residence.

Data were collected on patient demographics (age, gender, usual place of residence), pre-injury comorbidities and medications, mechanism of injury, admission vital signs, injuries and the need for critical care admission (Level 3). Preinjury polypharmacy was defined as five or more regular medications [21]. Traumatic Brain Injury (TBI) was defined as a head abbreviated injury score (AIS) ≥3 and injury severity was calculated using the Injury Severity Score (ISS) [22].

### Data sources / measurement

To compare feasibility and accuracy of ED frailty assessment three tools with potential utility in major trauma patients were chosen. Tools were selected by an expert panel of clinicians including ED and trauma specialists, geriatricians, nursing staff and patients based clinical application to an emergency setting. Tools were considered feasible for use in the ED if they were able to be fully completed using the information available at time of assessment, and accurate if there was agreement with the ‘gold standard’ of a geriatrician assessment of frailty (Additional file 1).

1. Trauma Specific Frailty Index (TSFI) is a scale composed of 15 variables designed to predict the presence of frailty in the trauma setting. It requires knowledge of functional state and pre-existing medical conditions. A TSFI score of > 0.27 is found to be an independent predictor of unfavourable outcomes after trauma [23].
2. Program of Research to Integrate Services for the Maintenance of Autonomy 7 (PRISMA7) is a self-report questionnaire comprising of 7 unambiguous questions aimed at identifying frail older adults. It utilises closed questions, ‘yes’ or ‘no’ answers, and a score of three or more is indicative of frailty [24].
3. Clinical Frailty Scale (CFS) is a 9-point scale using patient report or clinical judgement to assess functional capacity. It uses nine pictorial representations alongside a short descriptor to assign a frailty score: 1 (very fit) to 9 (terminally ill). Participants scoring 5 or more are considered frail [25].

Clinical and research nurses were trained in the use of each tool. Frailty assessment was performed in the ED using information available from the patient and/or carer, medical records, and clinical judgement. The geriatrician assessment was performed by a Consultant (Attending) or Specialist Registrar within 72 h of admission to hospital using CFS or as part of the Comprehensive Geriatric Assessment.

### Sample size

Prior data from the London Major Trauma system suggested that frailty affects 37% of major trauma patients aged 65 years and over. Based on this, the estimated number of patients required was 372, with 97% probability to achieve a 10% width of 95% confidence interval as the desired level of precision.

### Statistical analysis

Data were analysed using Stata (version 16.1). Comparisons of continuous data in frail and non-frail patients were conducted using t-tests. Due to the differing variables and defining scores in each tool we applied a dichotomous frailty measure (frail or non-frail) based on the clinically recommended scores in each of the tools. Analysis of categorical data was conducted using Fisher’s exact test. All tests are two-sided. Kappa statistic was used to measure the interrater agreement between ED assessed frailty according to TSFI, PRISMA7 and CFS with that of the geriatricians. A *p*-value of < 0.05 is considered statistically significant, and its corresponding false discovery rate using the Benjamini–Hochberg method is reported to account for multiplicity.

### Patient and public involvement

Members of a patient and public research expert group were involved in the design of the study and provided advice on consent procedures, content of the patient information material, and on acceptability (timing in the ED and number) of the frailty assessment tools used.

## Results

**Fig. 1:**
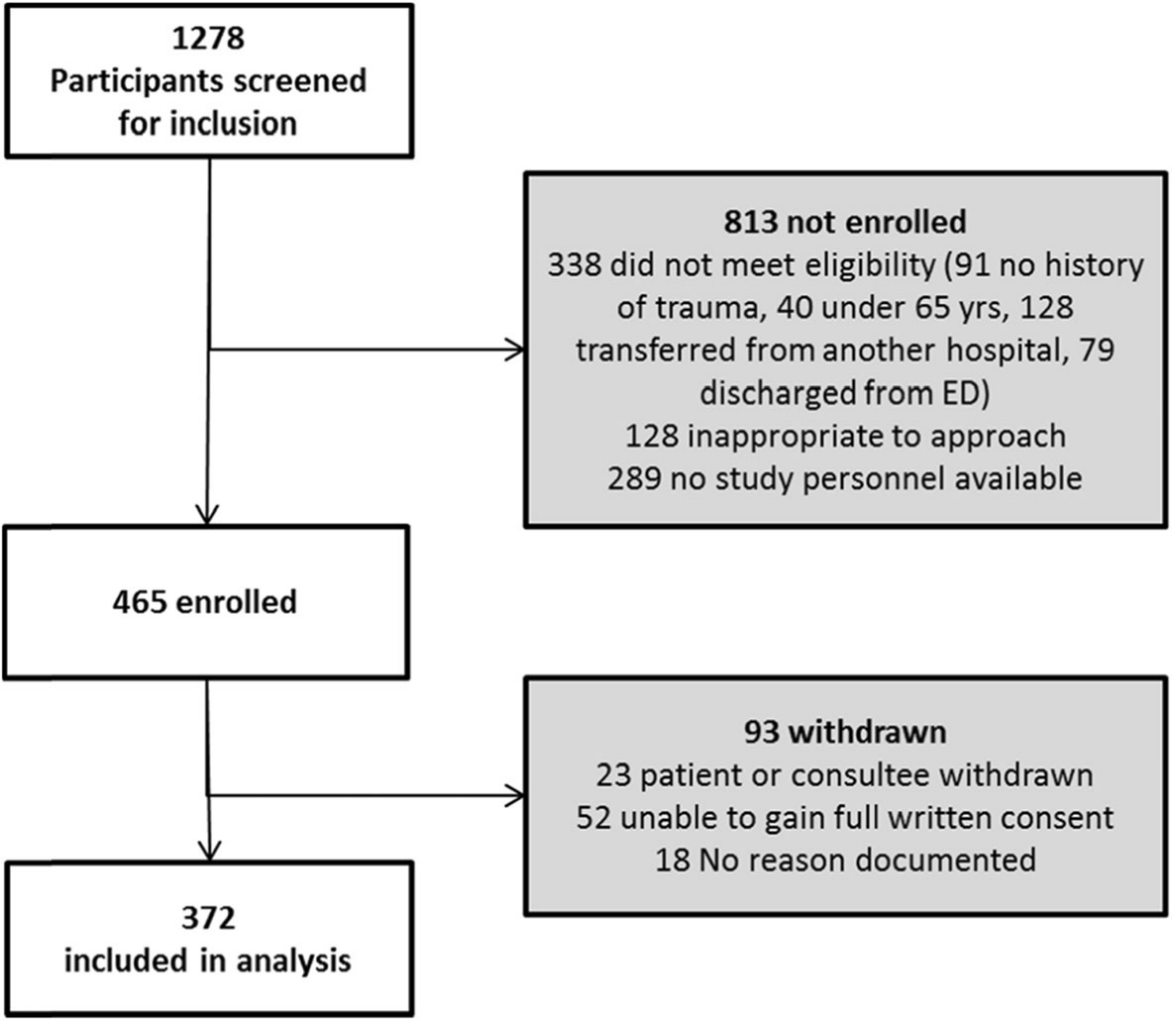
Flowchart of recruitment

A total of 1278 patients aged 65 or older admitted to hospital following major trauma were screened for inclusion into the study, 813 where not enrolled (Fig. 1). Of the remainder, 93 were withdrawn after enrolment as they declined to give consent, did not have a consultee or lacked capacity leaving 372 patients enrolled into the study.

The median age of the cohort was 80 years, more than half of patients were female and the majority lived in their own homes prior to the injury (Table 2). On average, patients had two pre-existing comorbidities and over a third took more than five regular medications daily. Low-level falls from less than 2 m were the leading mechanism of injury (56.7%) and one fifth of patients sustained a TBI (20.6%). A minority of patients were admitted to critical care as a result of their injuries (10.7%). Deaths in hospital occurred in 9.4% of cases and more than half of survivors were able to return to their usual place of residence from the MTC (Table 2).

**Table 2:** Demographic and clinical characteristics

n	372
Age, years, (median, IQR)	80 (73–86)
Female (n,%)	200 (53.8)
**Pre-admission residential status:**	
*Own home (n,%)*	338 (90.9)
*Residential facility with nursing (n,%)*	16 (4.3)
*Residential facility without nursing (n,%)*	11 (3.0)
*Warden controlled accommodation (n,%)*	6 (1.6)
*Unknown (n,%)*	1 (0.3)
Comorbidities, (median, IQR)	2 (1–3)
**Number of pre-injury medications**	
*1–5 (n,%)*	189 (50.8)
*> 5 (n,%)*	139 (37.4)
Predominant mechanisms of injury:	
*Fall < 2 m (n,%)*	211 (56.7)
*Fall > 2 m (n,%)*	79 (21.2)
*Pedestrian* vs *vehicle (n,%)*	36 (9.7)
Admission SBP mmHg, (median, IQR)	145 (125–166)
Admission GCS, (median, IQR)	15 (14–15)
TBI (n,%)	77 (20.6)
ISS, median (IQR)	16 (9–21)
Critical care (n,%)	40 (10.7)
**Outcomes**	
In-hospital mortality (n,%)	35 (9.4)
Critical care stay, days, (mean, SD)	1.06 (5.2)
Total MTC LOS, days, (median, IQR)	12 (5–20)
Discharge to usual place of residence (n,%)	210 (56.5)

*SBP* Systolic Blood Pressure, *GCS* Glasgow Coma Scale, *TBI* Traumatic Brain Injury, *ISS* Injury Severity Score, *MTC* Major Trauma Centre, *LOS* Length of Stay. Missing data: SBP: 4, GCS: 2, ISS: 39, LOS: 35

**Fig. 2:**
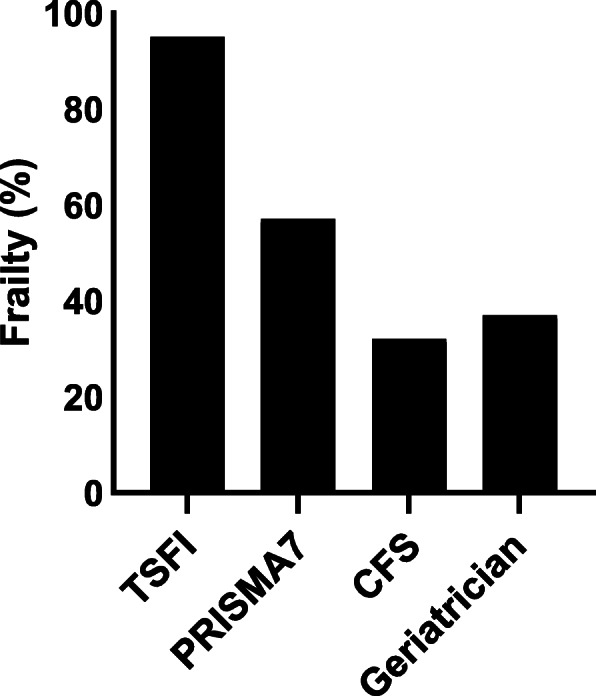
Bar graph represents proportion of frailty according to each tool: TSFI (Trauma Specific Frailty Index): 95%; PRISMA7 (Program of Research to Integrate Services for the Maintenance of Autonomy): 57%; CFS (Clinical Frailty Scale): 32%; GA (Geriatrician Assessment): 37%

The completion of frailty screening tools in the ED was variable. TSFI was completed in 31.9% of patients, compared to 93% with PRISMA7 and 98.9% with the CFS. Incidence of frailty also differed between tools. In patients with completed scores the TSFI identified the highest proportion of frail patients (95.0%), whereas just over half of the patients were frail according to PRISMA7 (57.1%) and a third with the CFS (31.8%). Of the 279 patients assessed by a geriatrician within 72 h of admission, 104 (37.2%) were considered to be frail (Fig. 2). Inter-rater agreement between the identification of frailty in the ED and that of the GA differed between tools. There was substantial agreement between CFS defined frailty and GA defined frailty (Kappa 0.637, *p* < 0.001). The agreement between PRISMA7 and the GA was moderate (Kappa 0.458, *p* < 0.001) but between the TSFI and GA agreement was slight (Kappa 0.103, *p* = 0.017).

Irrespective of screening tool, frail patients were significantly older than those deemed to be non-frail (*p* = 0.0012 for TSFI; *p* < 0.001 for others). PRISMA7 and CFS frail patients had a greater number of comorbidities compared to non-frail (*p* < 0.001) and were less likely to live in their own home prior to their injury (*p* < 0.05). Those taking more than five pre-injury medications were more likely to be frail across all tools (*p* < 0.05) but in higher proportions with PRISMA7 and CFS (Table 3). The incidence of falls < 2 m was greater in frail patients, with significantly higher rates in PRISMA7 (69.8%) and CFS (75.2%) cohorts compared to TSFI (54.9%). Rates of TBI and severity of injury did not differ significantly between frail and non-frail groups across the tools. Frail patients were significantly less likely to be admitted to critical care in all screening groups (*p* < 0.001). The greatest difference was observed in TSFI frailty where there was an 11-fold decrease in critical care admission for frail patients (66.6% vs. 6.1%, *p* < 0.001). Of the frail patients admitted to critical care, the stay length was shorter in all screening tool groups with the largest difference seen in the TSFI cohort (Non-Frail 10.5 days vs. Frail 0.64 days *p* < 0.001).

**Table 3:** Characteristics and outcomes per frailty tool groups (n = 372)

	TSFI Non-Frail	TSFI Frail	PRISMA7 Non-Frail	PRISMA7 Frail	CFS Non-Frail	CFS Frail
n (%)	6 (4.2)	113 (95.0)	147 (42.4)	199 (57.1)	251 (68.2)	117 (31.8)
Age, years (median, IQR)	69 (67–71)	81 (74–86)**	75 (70–80)	84 (77–89)**	78 (71–82)	87 (81–91)**
Female (n,%)	1 (16.7)	60 (53.1)	76 (51.7)	107 (53.8)	117 (46.6)	79 (67.5)
Pre-admission residential status:						
*Own home* (n,%)	5 (83.3)	104 (92.0)	142 (96.6)	174 (87.4)*	238 (94.8)	96 (82.1)**
*Residential facility with nursing* (n,%)	0 (0.0)	5 (4.4)	1 (0.7)	13 (6.5)	2 (0.8)	14 (12.0)
*Residential facility without nursing* (n,%)	1 (16.7)	3 (2.7)	3 (2.0)	7 (3.5)	7 (2.8)	4 (3.4)
*Warden controlled accommodation* (n,%)	0 (0.0)	1 (0.9)	1 (0.7)	5 (2.5)	3 (1.2)	3 (2.6)
*Unknown* (n,%)	–	–	–	–	1 (0.4)	0 (0.0)
Comorbidities (median, IQR)	1 (1–3)	2 (1–3)	2 (1–3)	3 (2–4)**	2 (1–3)	3 (2–4)**
> 5 pre-injury medications (n,%)	0 (0.0)	46 (40.7)*	30 (20.4)	101 (50.8) **	70 (27.9)	69 (59.0) **
Predominant Mechanism of Injury:						
*Fall < 2 m* (n,%)	0 (0.0)	62 (54.9)*	62 (42.2)	139 (69.8)**	123 (49.0)	88 (75.2)**
*Fall > 2 m* (n,%)	4 (66.7)	27 (23.9)	39 (26.5)	32 (16.1)	57 (22.7)	19 (16.2)
*Pedestrian* vs *vehicle* (n,%)	0 (0.0)	14 (12.4)	21 (14.3)	13 (6.5)	31 (12.4)	5 (4.3)
Admission SBP mmHg (median, IQR)	124 (96–149)	143 (129–162)	139 (120–164)	148 (130–170)*	141 (120–164)	150 (133–170)**
Admission GCS (median, IQR)	15 (15–15)	15 (14–15)	15 (14–15)	15 (14–15)	15 (14–15)	15 (14–15)
TBI (n,%)	0 (0.0)	7 (6.1)	15 (10.2)	24 (12.0)	26 (10.3)	17 (14.5)
ISS (median, IQR)	23 (20–29)	17 (9–26)	16 (9–22)	13 (9–20)	16 (9–22)	13 (9–20)
Critical Care (n,%)	4 (66.6)	7 (6.1)**	24 (16.3)	10 (5.0)**	37 (14.7)	2 (1.7)**
*Outcomes*						
In-hospital mortality (n,%)	0 (0)	12 (10.6)	5 (3.4)	27 (13.6)*	15 (6)	20 (17.1)*
Critical care stay, days (mean, SD)	10.5 (17.9)	0.64 (4.22)**	1.8 (6.2)	0.55 (4.6)*	1.5 (6.3)	0.03 (0.29)*
Total MTC LOS, days (median, IQR)	24 (8–28)	9 (3–19)	12 (4–21)	12 (5–19)	12 (4–20)	13 (6–21)
Discharge to usual place of residence (n,%)	2 (33.3)	62 (62.8)	106 (75.6)	119 (69.1)	169 (71.6)	66 (68.0)

*MOI* Mechanism of Injury, *SBP* Systolic Blood Pressure, *GCS* Glasgow Coma Scale, *TBI* Traumatic Brain Injury, *ISS* Injury Severity Score, *MTC* Major Trauma Centre, *LOS* Length of Stay. ** *p* ≤ 0.001; * *p* < 0.05 comparing non-frail and frail groups (t-test for continuous variables; Fisher’s exact test for categorical variables). False discovery rate = 0.066. Missing data: TSFI Frail ISS:50; PRISMA7 Non-Frail: ISS: 71, LOS: 5; PRISMA7 Frail: SBP: 1, GCS: 1, ISS: 78, LOS: 27; CFS Non-Frail: SBP: 3, GCS: 1, ISS: 92, LOS: 15; CFS Frail: GCS: 1, ISS: 46, LOS: 20

**Fig. 3:**
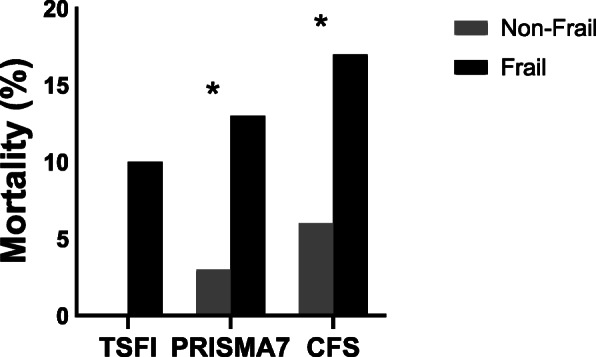
Bar graph represents mortality for non-frail and frail cohorts (*n* = 372). TSFI non-frail 0% vs. frail 10% *p* = 1.000; PRISMA7 non-frail 3% vs. frail 13% *p = 0.001; CFA non-frail 6% vs. frail 17% *p = 0.002

Mortality was greatest in all frail cohorts but rates differed between screening tool groups (Fig. 3). TSFI frailty had the lowest proportion of deaths (Non-frail 0% vs. Frail 10.6%, *p* = 1.000) compared to PRISMA7 (Non-frail 3.4% vs. Frail 13.6%, *p* = 0.001) and CFS (Non-frail 6.0% vs. Frail 17.1%, *p* = 0.002) respectively. Irrespective of tool, there were few differences between frail and non-frail groups for critical care and hospital length of stay or discharge back to their previous place of residence (Table 3).

## Discussion

This prospective study has demonstrated that screening for frailty in older major trauma patients within the ED is feasible and accurate. However this appears to depend on which tool is used and our results suggest that frailty determined by the Clinical Frailty Scale had the strongest agreement with specialist geriatrician assessment. Frailty was associated with increased age and previously defined characteristics of frail syndromes were best identified by PRISMA7 and CFS. Critical care resource use differed between frail and non-frail patients, and mortality was increased in those identified as frail, greatest in the CFS defined group.

The three tools used within this study represent different approaches to the ED assessment of frailty in major trauma patients. Each of the screening tools needs to be completed fully for a score to be derived and there were significant differences in the completion rates across tools. The 15-point TSFI completion rate was less than 32%, rising to 93% (PRISMA7) and nearly 99% (CFS). Studies that compare the ability of different frailty tools to prognosticate or identify resource use do not report completion rates as these patients are usually excluded from any analysis. Measurement of frailty in the ED is known to be challenging as the information required to make the assessment may not be available or the patient’s clinical condition may leave them unable to answer questions directly. Previous studies have identified the optimal characteristics of frailty tools appropriate for use in ED patients as those which can be applied quickly, do not require the use of complex equipment and use objective parameters [26–28]. The variation in the levels of completion could be explained by the degree of complexity in the tools. The TSFI requires knowledge of social history and physical and sexual activity that may not be readily available or appropriate to question in the acute phases of trauma care, whereas CFS relies on patient report or clinical judgement to assess a single indicator (functional capacity). The low completion rate of the TSFI in this study indicates that it is not a feasible tool to use in the ED phase of older trauma management.

Screening using the different tools resulted in a wide variation in the percentage of patients identified as frail compared to the ‘gold standard’ geriatrician assessment of frailty. We used dichotomous scores (frail / not frail) to allow for comparison across tools and a greater proportion of patients were assessed as frail using the TSFI compared to the PRISMA7 and CSF. Our findings differ from previous reports in major trauma patients age 65 or over with prevalence of frailty ranging from 14 to 44% dependent on the tool used [11, 23]. However, direct comparison of frailty prevalence across existing studies is hampered by the large number of tools reported and the differences in their application (assessor type, comparator, timing of assessment).

Frailty defined by CFS in our study was similar to that in other major trauma studies using the Clinical Frailty Scale [29, 30]. However we found that ED CFS had the strongest agreement with geriatrician assessed frailty, whereas a recent study of patients with medical conditions only observed a weak agreement between ED clinical frailty scale assessment and that of in-hospital physicians [31]. PRISMA7 frailty was higher than in other studies of older major trauma patients but showed moderate agreement between the ED and geriatrician scores [23, 28, 29]. PRISMA7 use in major trauma patients is unreported although in a recent non-selective ED population was found to have a higher accuracy in separating frail from non-frail compared to CFS and Identification of Seniors at Risk Tool [32]. Whilst TSFI identified the greatest proportion of patients as being frail it had the weakest agreement with GA. The tool relies less on clinical judgement and more on objective measures in comparison to CFS, which should make it more accurate in differentiating frail from non-frail trauma patients, but was not the case in our study. We are unable to account for the high rates of frailty scored using TSFI, which were over twice or three times that found in other older trauma in-patient populations [23, 30, 33].

In line with other studies, frailty was characterised by increased age, comorbidity, polypharmacy and low level falls across all tools in this study [25, 33–36]. Frail patients were less severely injured across the cohorts and this may be due to lower energy mechanisms associated with reduced mobility and activity levels. However the lower ISS observed in frail patients may have implications for the levels of geriatric specialist input required. A recent single site study of geriatrician-defined frailty reported concerns that frail patients with an ISS < 15 would not qualify for the best practice payment given in UK and the financial incentive to support geriatrician review would be lost [29]. In our multi-site study the median ISS in both PRISMA7 and CFS frail groups fell below the ISS severe injury definition and may not have been triggered a geriatrician review. This underpins the need for accurate, early identification of frailty to ensure ED initiated frailty specialist pathways in older trauma patients, irrespective of ISS.

Whilst mortality differed across tools, rates were similar for those reported in other frail trauma populations [29, 37, 38]. The greatest proportion of deaths were in those who were frail according to CSF and PRISMA7, which may mean these tools identified the frailest patients. Frailty is a predictor of mortality in older trauma patients [29, 37, 39]. Our findings suggest that ED assessment using CFS may enable early specialist geriatric pathways to improve outcomes and enhance survival.

Whilst overall hospital length of stay and home discharge did not differ across groups, the proportion of patients identified as frail were less likely to be admitted to critical care. In those that were, the length of stay for survivors was shorter than for the non-frail group. It is not clear from our work why the admission rate to critical care is low for the majority of frail patients but a similar trend was reported in older major trauma patients with an ISS > 15 [7, 40]. It may be due to appropriate step down from critical care to ward-based care for patients following a period of optimization or where a ceiling of care is identified.

### Limitations

This study has a number of limitations. TSFI was completed in just under a third of the patients and this small sample size may have affected statistical power and may not accurately reflect the utility of this tool in non ED settings. In addition, this study only looks at simple associations between frailty and various factors using unadjusted analysis without controlling confounding factors, so the results do not indicate causal relationships. If the geriatricians assessed frailty using a tool then CFS was utilised, which may have positively influenced the agreements with ED CFS assessment. However the expert geriatrician ‘diagnosis’ of frailty was deemed to be the gold standard within this study. Ideally the geriatricians would have assessed all of the patients with all of the tools however this was not feasible within geriatric clinical workloads.

Formal validation was not performed however bias was minimised using standardised measures and the provision of consistent face to face training with the nurses on the use of the tools.

We acknowledge that older trauma patients are also admitted to Trauma Units (Level 2–3 hospitals) and findings in these settings may differ to that of an MTC.

Finally, whilst this study was conducted in the UK our findings may not be applicable to all emergency health settings, although may be of interest to those with similar trauma systems.

## Conclusions

Our findings suggest that the CFS is the most suitable screening tool to identify frailty in older major trauma patients in the ED when compared to both the PRISMA7 and TSFI tools. The results provide evidence that the CFS is reliable and feasible to complete early in the major trauma patient’s pathway prior to admission to an in-patient area. We provide further evidence of agreement between ED nurses and subsequent physician assessment of frailty suggesting CFS can be used to distinguish between frail and non-frail major trauma patients in the ED [41]. We propose that as the largest healthcare workforce within major trauma care that nursing staff are ideally placed at the bedside to identify frailty early in this patient group. This early identification of frailty should be followed by improved frailty specific clinical pathways and interventions that positively impact on health and longer term recovery.

## Supplementary Information

**Additional file 1.**

## Data Availability

The datasets used and/or analysed during the current study are available from the corresponding author on reasonable request.

## Abbreviations

AIS: Abbreviated Injury Scale
CFS: Clinical Frailty Scale
ED: Emergency Department
GCS: Glasgow Coma Scale
ISS: Injury Severity Score
LOS: Length of Stay
MTC: Major Trauma Centre
PRISMA7: Program of Research to Integrate Services of Autonomy
TBI: Traumatic Brain Injury
TSFI: Trauma specific frailty index
