# Supplementary material for: Feasibility and accuracy of ED frailty identification in older trauma patients: a prospective multi-centre study
Source: Scand J Trauma Resusc Emerg Med. 2021 Mar 30;29:54. doi: 10.1186/s13049-021-00868-4 (PMC8011126; doi:10.1186/s13049-021-00868-4)
Supplement: Supplementary file 1 — Additional file 1. [file 13049_2021_868_MOESM1_ESM.pdf]

CASE REPORT FORM

FRAIL-T

The frailty in major trauma study

Version 0.3

Date 26 12 2018

PARTICIPANT INITIALS ☐☐☐

PARTICIPANT NUMBER ☐☐☐/☐☐☐☐ Site / Participant number

DATE ☐☐/☐☐/☐☐☐☐

AGE ☐☐

SEX FEMALE ☐ MALE ☐

ETHNICITY

	Code (circle)
Asian – any other Asian background	00
Asian or Asian British - Bangladeshi	01
Asian or Asian British – Indian	02
Asian or Asian British – Pakistanis	03
Black – any other black background	04
Black or Black British – African	05
Black or Black British – Caribbean	06
Mixed – any other mixed background	07
Mixed – white and Asian	08
Mixed – white and Black African	09
Mixed – whist and Black Caribean	10
Other – any other ethnic group	11
Other – Chinese	12
Other – not known	13
Other – not stated	14
White – any other white background	15
White – British	16
White – Irish	17

PARTICIPANT INITIALS ☐☐☐ PARTICIPANT NUMBER ☐☐☐/☐☐☐☐

CASE REPORT FORM

INJURY AND CLINICAL CHARACTERISTICS

Date of ED attendance		Time of ED attendance
☐☐/☐☐/☐☐☐☐		(HH:MM): ☐
Trauma call		Time of trauma team arrival
Yes / No		(HH:MM): ☐
'Code Red'	Hospital trauma team	ED trauma team
Date of traumatic injury		Time of traumatic injury
☐☐/☐☐/☐☐☐☐		(HH:MM): ☐
Location of traumatic injury – postcode		Location of patient's home – postcode
☐☐☐☐		☐☐☐☐
Type of injury Blunt ☐		Penetrating ☐

Mechanism of Injury	Code (circle)	Mechanism of Injury	Code (circle)
Pedestrian vs vehicle	01	Cycling incident	12
Motorcycle vs other vehicle	02	Sports injury	13
Car vs stationary object	03	Cyclist vs vehicle	14
Car vs vehicle	04	Motorcycle vs stationary object	15
Pedestrian vs motorcycle	05	Hit by train/tram	16
Pedestrian vs falling object	06	Crush injury	17
Stabbing	07	Blast injury	18
Fall from height	08	Deliberate self-harm	19
Blunt assault	09	Other	20
Jump from height	10	Fall <2m (e.g. low level fall from standing)	21
Gunshot wound	11		

ED VITAL SIGNS ON PRESENTATION (1ST SET OF ED OBSERVATIONS)

Glasgow Coma Score (GCS)	Systolic blood pressure (SBP): mmHg
E: V: M:	Temperature:
Heart rate:	1st lactate:

PARTICIPANT INITIALS ☐☐☐ PARTICIPANT NUMBER ☐☐☐/☐☐☐☐

PAST MEDICAL HISTORY

Comorbidities

Region	Comorbidity	Code (circle all that apply)
Cardiac	Angina, Arrhythmias, Heart failure, MI, Valve disease	01
Vascular	Stroke/TIA, Hypertension, Peripheral vascular disease	02
Respiratory	Asthma, COPD, emphysema	03
Neurological	Dementia, Hemiplegia/paraplegia, Degenerative disorders e.g. Parkinson	04
Endocrine	Type 1 or Type 2 Diabetes	05
Renal	Moderate or severe renal disease	06
GI	GORD, GI ulceration, Inflammatory bowel diseases, Liver disease	07
Cancer/immunity	HIV/AIDS, Active cancer, Leukaemia, Lymphoma, Metastatic disease	08
MSK	Rheumatoid or osteoarthritis, Osteoporosis, Connective tissue disorders	09
Psychological	Depression, Anxiety disorders, Bipolar disease, Schizophrenia/psychosis	10
Substances	Alcohol dependence, drug use, smoker	11
Senses	Significant hearing or visual impairment	12
Other	Any other significant comorbidity (deemed by research or clinical team)	13

If other, please enter details:

Number of regular significant pre-injury medications (not paracetamol, lactulose for example)		
None	1-5	More than 5

Pre-admission residential status

	Code (circle)
Homeless in night shelter	00
Homeless without accommodation	01
Medical area	02
Not given; patient physically unable	03
Not given; patient refused	04
Not known	05
Own stable accommodation e.g. home or flat	06
Residential WITH routine nursing care	07
Residential WITHOUT routine nursing care	08
Warden controlled accommodation	09

Discharge location from ED

	Code (circle)
Ward (under major trauma / surgical team)	00
Ward (under medical team)	01
Ward (under geriatric team)	02
HDU	03
ITU	04
Died in ED	05

PARTICIPANT INITIALS ☐☐☐ PARTICIPANT NUMBER ☐☐☐/☐☐☐☐

NURSE-LED FRAILTY ASSESSMENT

Initials of person completing assessments	☐☐☐

CLINICAL FRAILTY SCALE (CFS)

Are you able to fully complete this assessment? Circle	Yes	No
If no, please explain why not?		

Where did you get the information to complete this assessment? Circle all that apply				
Patient	Relative	Pre-hospital information	Medical records	Own judgment

Please circle the measurement on the scale below:

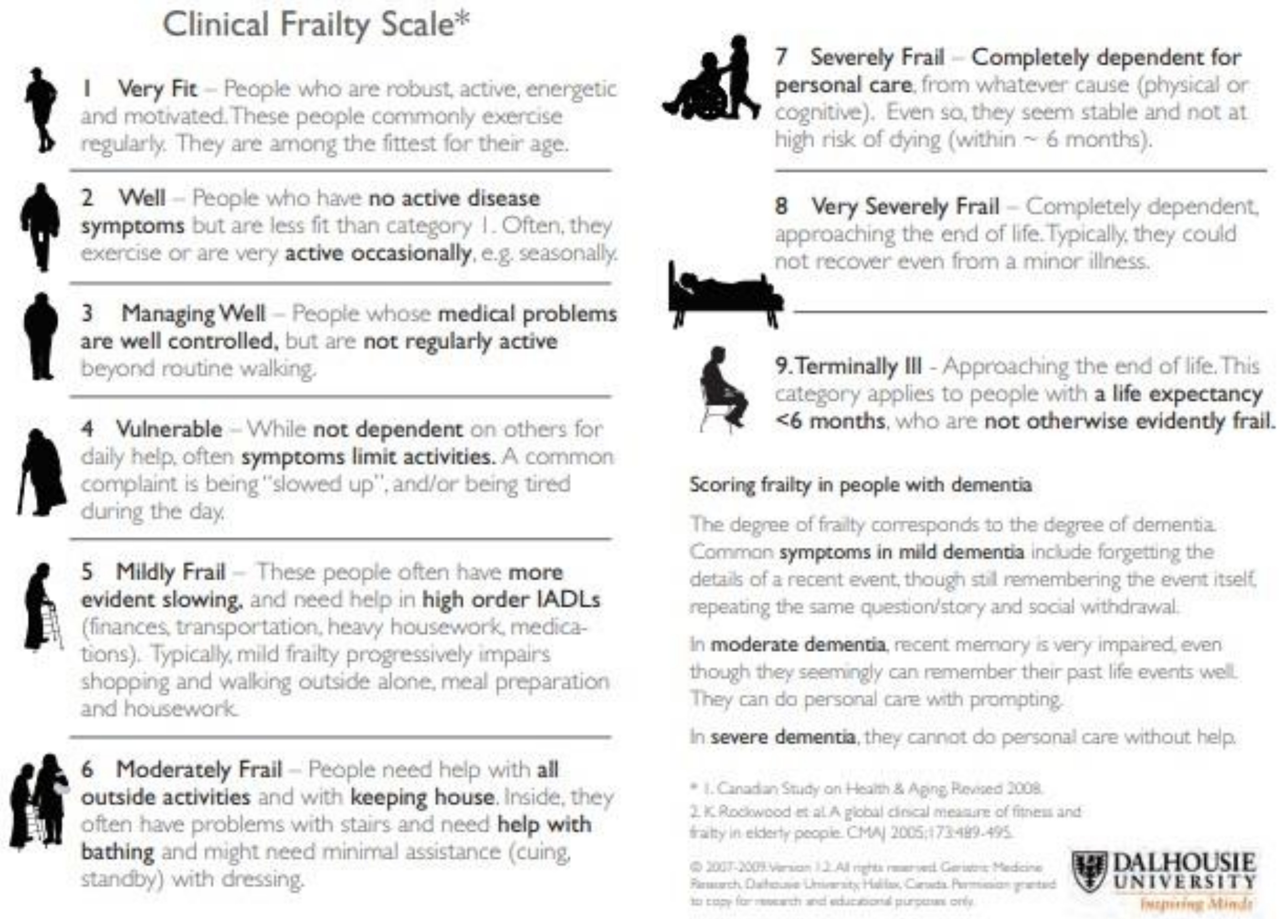

Clinical Frailty Scale*

1 Very Fit – People who are robust, active, energetic and motivated. These people commonly exercise regularly. They are among the fittest for their age.

2 Well – People who have **no active disease symptoms** but are less fit than category 1. Often, they exercise or are very **active occasionally**, e.g. seasonally.

3 Managing Well – People whose **medical problems are well controlled**, but are **not regularly active** beyond routine walking.

4 Vulnerable – While **not dependent** on others for daily help, often **symptoms limit activities.** A common complaint is being "slowed up", and/or being tired during the day.

5 Mildly Frail – These people often have **more evident slowing**, and need help in **high order IADLs** (finances, transportation, heavy housework, medications). Typically, mild frailty progressively impairs shopping and walking outside alone, meal preparation and housework.

6 Moderately Frail – People need help with **all outside activities** and with **keeping house**. Inside, they often have problems with stairs and need **help with bathing** and might need minimal assistance (cuing, standby) with dressing.

7 Severely Frail – **Completely dependent for personal care**, from whatever cause (physical or cognitive). Even so, they seem stable and not at high risk of dying (within ~ 6 months).

8 Very Severely Frail – Completely dependent, approaching the end of life. Typically, they could not recover even from a minor illness.

9. Terminally Ill - Approaching the end of life. This category applies to people with **a life expectancy <6 months**, who are **not otherwise evidently frail.**

Scoring frailty in people with dementia

The degree of frailty corresponds to the degree of dementia. Common **symptoms in mild dementia** include forgetting the details of a recent event, though still remembering the event itself, repeating the same question/story and social withdrawal.

In **moderate dementia**, recent memory is very impaired, even though they seemingly can remember their past life events well. They can do personal care with prompting.

In **severe dementia**, they cannot do personal care without help.

* 1. Canadian Study on Health & Aging, Revised 2008.
2. K. Rockwood et al. A global clinical measure of fitness and frailty in elderly people. CMAJ 2005;173:489-495.

© 2007-2009. Version 1.2. All rights reserved. Geriatric Medicine Research, Dalhousie University, Halifax, Canada. Permission granted to copy for research and educational purposes only.

DALHOUSIE UNIVERSITY Inspiring Minds

PARTICIPANT INITIALS ☐☐☐ PARTICIPANT NUMBER ☐☐☐/☐☐☐☐

PRISMA-7

Are you able to fully complete this assessment? Circle	Yes	No
If no, please explain why not?		

Where did you get the information to complete this assessment? Circle all that apply				
Patient	Relative	Pre-hospital information	Medical records	Own judgment

PRISMA-7 Questionnaire

PATIENT QUESTIONS		
1. Are you older than 85 years?	Yes ☐	No ☐
2. Are you male?	Yes ☐	No ☐
3. In general, do you have any health problems that require you to limit your activities?	Yes ☐	No ☐
4. Do you need someone to help you on a regular basis?	Yes ☐	No ☐
5. In general, do you have any health problems that require you to stay at home?	Yes ☐	No ☐
6. If you need help, can you count on someone close to you?	Yes ☐	No ☐
7. Do you regularly use a stick, walker or wheelchair to move about?	Yes ☐	No ☐
**Total checked:**	_____	_____

Instructions:

- For questions 3 through 7, do not interpret the answer; simply note the person's answer without considering whether or not it should be "yes" or "no".
- If the respondent hesitates between "yes" and "no", ask him/her to choose one of the two answers.
- If, despite several attempts, he/she persists in answering "a little" or "at times", enter "yes".

SCORING: If the respondent had 3 or more "yes" answers, this indicates an increased risk of frailty and the need for further clinical review.

PARTICIPANT INITIALS ☐☐☐ PARTICIPANT NUMBER ☐☐☐/☐☐☐☐

15 VARIABLE TRAUMA SPECIFIC FRAILTY INDEX

Are you able to fully complete this assessment? Circle	Yes	No
If no, please explain why not?		

Where did you get the information to complete this assessment? Circle all that apply

Patient	Relative	Pre-hospital information	Medical records	Own judgment

Please score each line (number in brackets) on the measurement to produce a total score.

**Comorbidities**			
Cancer history	Yes (1)		No (0)
Coronary Heart Disease (more than one score possible)	MI (1)		CABG (0.75)
	PCI (0.5)		Medication (0.25)
Dementia	Severe (1)		Moderate (0.5)
**Daily activities**			
Help with grooming	Yes (1)		No (0)
Help with managing money	Yes (1)		No (0)
Help doing household work	Yes (1)		No (0)
Help toileting	Yes (1)		No (0)
Help walking	Wheelchair (1)		Walker / frame (0.75)
	Cane (0.25)		No (0)
**Health Attitude**			
Feel less useful	Most time (1)	Sometimes (0.5)	Never (0)
Feel sad	Most time (1)	Sometimes (0.5)	Never (0)
Feel effort to do everything	Most time (1)	Sometimes (0.5)	Never (0)
Falls	Most time (1)	Sometimes (0.5)	Never (0)
Feel lonely	Most time (1)	Sometimes (0.5)	Never (0)
**Function**			
	No (1)		Yes (0)
**Nutrition**			
Albumin	< 30 g/L (1)		> 30 g/L (0)

TOTAL ____________

NURSE-LED FRAILTY ASSESSMENT – EVALUATION OF TOOLS

Please rank the tools from **best to worst.**<br>1 = best<br>2 = middle<br>3 = worst	**Clinical Frailty Scale**<br><br>Number: _______	**PRISMA-7**<br><br>Number: _______	**Trauma Specific Frailty Index**<br><br>Number: _______

	**Clinical Frailty Scale**	**PRISMA-7**	**Trauma Specific Frailty Index**
For each tool, please circle the ***number (rating)*** most applies for each tool	5 = extremely easy to complete<br><br>4 = somewhat easy to complete<br><br>3 = neither easy nor hard to complete<br><br>2 = hard to complete<br><br>1 = extremely hard to complete	5 = extremely easy to complete<br><br>4 = somewhat easy to complete<br><br>3 = neither easy nor hard to complete<br><br>2 = hard to complete<br><br>1 = extremely hard to complete	5 = extremely easy to complete<br><br>4 = somewhat easy to complete<br><br>3 = neither easy nor hard to complete<br><br>2 = hard to complete<br><br>1 = extremely hard to complete

PARTICIPANT INITIALS ☐☐☐ PARTICIPANT NUMBER ☐☐☐/☐☐☐☐

GERIATRICIAN ASSESSMENT WITHIN 72 HOURS

Date of assessment ☐☐/☐☐/☐☐☐☐		
Designation of geriatrician completing assessment. Circle	ST3 +	Consultant
Was frailty present in this patient's pre-trauma state? Circle	Yes	No

Please circle the measurement on the scale below:

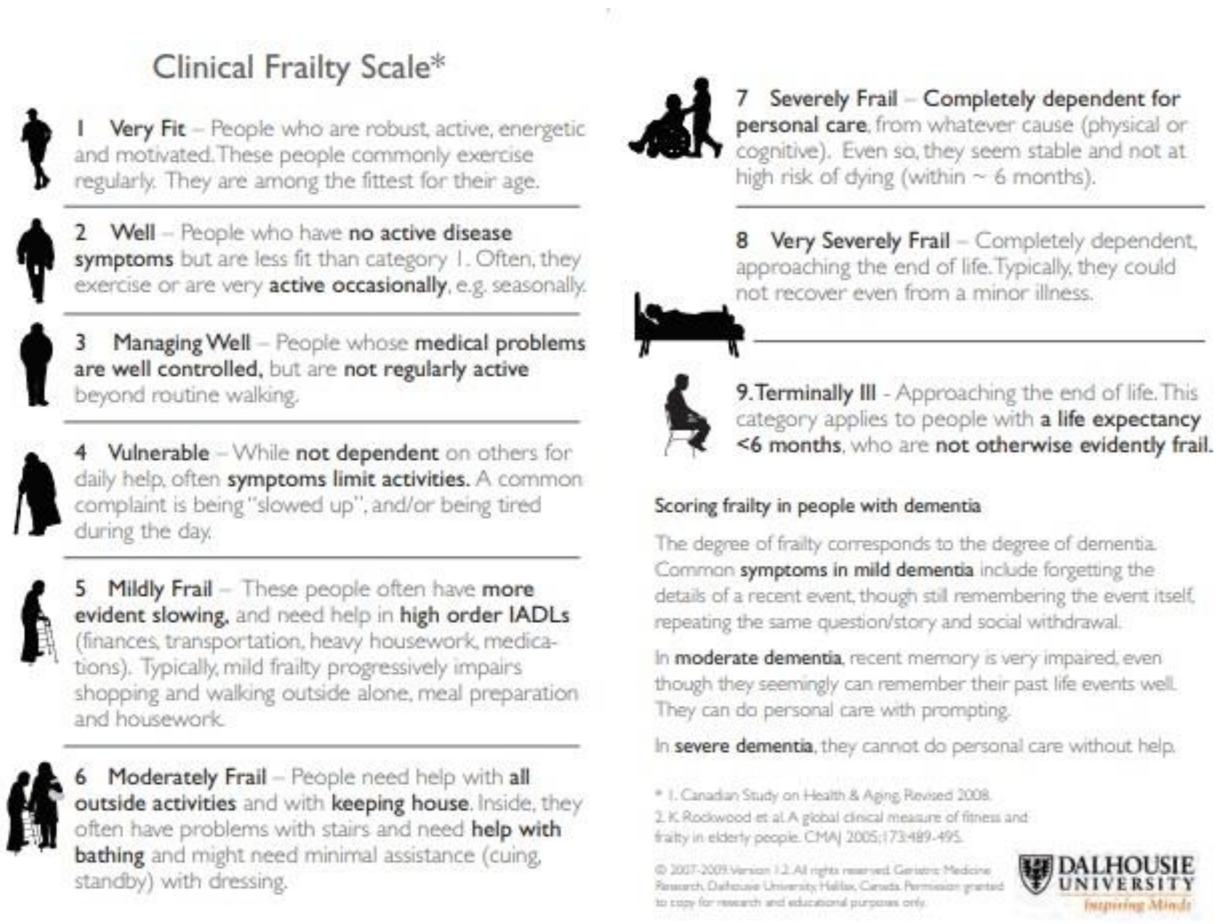

Clinical Frailty Scale*

1 **Very Fit** – People who are robust, active, energetic and motivated. These people commonly exercise regularly. They are among the fittest for their age.

2 **Well** – People who have **no active disease symptoms** but are less fit than category 1. Often, they exercise or are very **active occasionally**, e.g. seasonally.

3 **Managing Well** – People whose **medical problems are well controlled,** but are **not regularly active** beyond routine walking.

4 **Vulnerable** – While **not dependent** on others for daily help, often **symptoms limit activities.** A common complaint is being "slowed up", and/or being tired during the day.

5 **Mildly Frail** – These people often have **more evident slowing,** and need help in **high order IADLs** (finances, transportation, heavy housework, medications). Typically, mild frailty progressively impairs shopping and walking outside alone, meal preparation and housework.

6 **Moderately Frail** – People need help with **all outside activities** and with **keeping house.** Inside, they often have problems with stairs and need **help with bathing** and might need minimal assistance (cuing, standby) with dressing.

7 **Severely Frail** – **Completely dependent for personal care,** from whatever cause (physical or cognitive). Even so, they seem stable and not at high risk of dying (within ~ 6 months).

8 **Very Severely Frail** – Completely dependent, approaching the end of life. Typically, they could not recover even from a minor illness.

9. **Terminally Ill** - Approaching the end of life. This category applies to people with **a life expectancy <6 months,** who are **not otherwise evidently frail.**

Scoring frailty in people with dementia

The degree of frailty corresponds to the degree of dementia. Common **symptoms in mild dementia** include forgetting the details of a recent event, though still remembering the event itself, repeating the same question/story and social withdrawal.

In **moderate dementia,** recent memory is very impaired, even though they seemingly can remember their past life events well. They can do personal care with prompting.

In **severe dementia,** they cannot do personal care without help.

* 1. Canadian Study on Health & Aging, Revised 2008.
2. K. Rockwood et al. A global clinical measure of fitness and frailty in elderly people. CMAJ 2005;173:489-495.

© 2007-2009. Version 1.2. All rights reserved. Geriatric Medicine Research, Dalhousie University, Halifax, Canada. Permission granted to copy for research and educational purposes only.

DALHOUSIE UNIVERSITY Inspiring Minds

OUTCOMES & FOLLOW UP: ON DISCHARGE FROM HOSPITAL

The Abbreviated Injury Scale. (AIS) Maximum AIS for region:	
AIS Head and neck	
AIS Face	
AIS Thorax	
AIS Abdo/pelvic contents/lumbar spine	
AIS extremity/bony pelvis	
AIS external	
ISS	

Date of discharge or death	Did the patient die?
☐☐/☐☐ /☐☐☐☐	Yes / No
Critical Care admission (L3) – length of stay (days)	Critical Care admission (L2) – length of stay (days)
☐☐☐☐	☐☐☐☐
Total length of stay (days)	
☐☐☐☐	

DISCHARGE DESTINATION

	Code (circle)
Same as previous (usual place of residence)	00
Homeless without accommodation	01
Medical area (other hospital)	02
Medical area (rehabilitation facility)	03
Not given; patient physically unable	04
Not given; patient refused	05
Not known	06
Own stable accommodation e.g. home or flat	07
Residential WITH routine nursing care	08
Residential WITHOUT routine nursing care	09
Warden controlled accommodation	10

PARTICIPANT INITIALS ☐☐☐ PARTICIPANT NUMBER ☐☐☐/☐☐☐☐

EQ-5D-5L

Date of completion	☐☐/☐☐/☐☐☐☐

Under each heading, please tick the **ONE** box that best describes your health **TODAY**

MOBILITY

I have no problems in walking about ❑
I have slight problems in walking about ❑
I have moderate problems in walking about ❑
I have severe problems in walking about ❑
I am unable to walk about ❑

SELF-CARE

I have no problems washing or dressing myself ❑
I have slight problems washing or dressing myself ❑
I have moderate problems washing or dressing myself ❑
I have severe problems washing or dressing myself ❑
I am unable to wash or dress myself ❑

USUAL ACTIVITIES *(e.g. work, study, housework, family or leisure activities)*

I have no problems doing my usual activities ❑
I have slight problems doing my usual activities ❑
I have moderate problems doing my usual activities ❑
I have severe problems doing my usual activities ❑
I am unable to do my usual activities ❑

PAIN / DISCOMFORT

I have no pain or discomfort ❑
I have slight pain or discomfort ❑
I have moderate pain or discom ❑
I have severe pain or discomfort ❑
I have extreme pain or discomfort ❑

ANXIETY / DEPRESSION

I am not anxious or depressed ❑
I am slightly anxious or depressed ❑
I am moderately anxious or depressed ❑
I am severely anxious or depressed ❑
I am extremely anxious or depressed ❑

PARTICIPANT INITIALS ☐☐☐ PARTICIPANT NUMBER ☐☐☐/☐☐☐☐

EQ-5D-5L

- We would like to know how good or bad your health is TODAY.
- This scale is numbered from 0 to 100.
- 100 means the best health you can imagine.
  0 means the worst health you can imagine.
- Mark an X on the scale to indicate how your health is TODAY.
- Now, please write the number you marked on the scale in the box below.

YOUR HEALTH TODAY = ☐

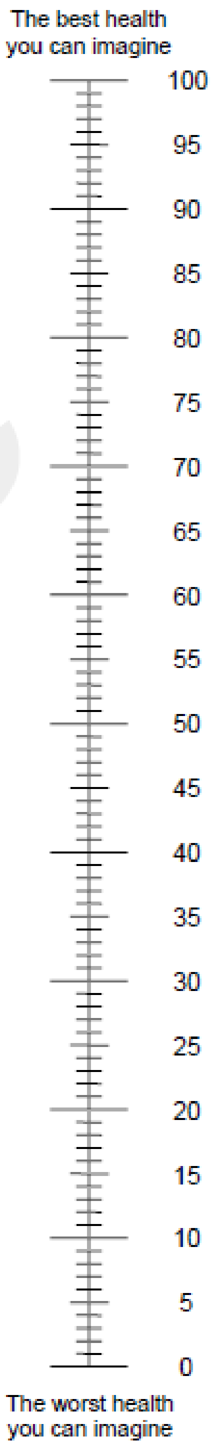

Sample

END OF CRF

PLEASE ENTER DATA INTO REDcap (web address)
